# Vulnerable Plaque, Characteristics, Detection, and Potential Therapies

**DOI:** 10.3390/jcdd6030026

**Published:** 2019-07-27

**Authors:** Anouar Hafiane

**Affiliations:** Department of Medicine, Faculty of Medicine and Research Institute of the McGill University, Health Centre, McGill University, Montreal, QC H4A 3J1, Canada; anouar.hafiane@mail.mcgill.ca or Tel.: +(514) 934-1934 (ext. 36012)

**Keywords:** plaque, atherosclerosis, regression, thrombosis, cardiovascular disease

## Abstract

Plaque development and rupture are hallmarks of atherosclerotic vascular disease. Despite current therapeutic developments, there is an unmet necessity in the prevention of atherosclerotic vascular disease. It remains a challenge to determine at an early stage if atherosclerotic plaque will become unstable and vulnerable. The arrival of molecular imaging is receiving more attention, considering it allows for a better understanding of the biology of human plaque and vulnerabilities. Various plaque therapies with common goals have been tested in high-risk patients with cardiovascular disease. In this work, the process of plaque instability, along with current technologies for sensing and predicting high-risk plaques, is debated. Updates on potential novel therapeutic approaches are also summarized.

## 1. Introduction

Ruptures of atherosclerotic plaques and consequent acute cardiovascular complications are still the leading causes of morbidity and mortality worldwide [1,2]. Incidences of this disease are continuing to rise despite recent advances in primary prevention and therapeutic technology [1,3,4]. Atherosclerosis is a smoldering immuno-inflammatory illness powered by lipids [5], where atherosclerotic plaque is considered the hallmark of atherosclerosis lesions [3,5]. Inflammation plays an important role in plaque progression, where inflammatory tissue factors are key contributors to plaque thrombogenicity [6,7,8]. However, the mechanism of plaque rupture and thrombosis reflects complex biological dynamic cellular processes [9]. This event mostly involves various stages of cell modification, activation, and dysfunction [10]. Our knowledge of plaque biology is constantly expanding. Nowadays, despite the many possible targets for atherosclerosis, the best treatment choice is the use of high-intensity statins treatment [11]. In this review, the processes in decreased plaque stability are discussed for the detection and prediction of high-risk plaques along with current and experimental techniques. Finally, potential alternative therapeutic strategies are summarized.

## 2. Biology of Atheromatous Plaque Formation and Progression

**Figure 1:**
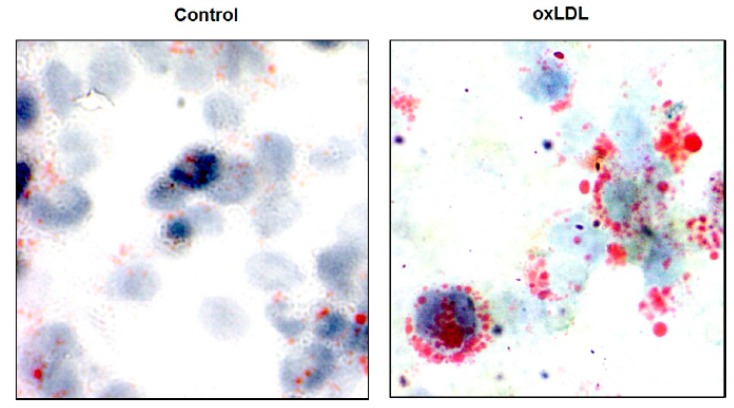
Foam cell formation. Foam cells were formed after the incubation of human monocyte derived from macrophages with 100 μg/mL of oxidized low-density lipoprotein (LDL) for 2 days. After fixation with formalin for 1 h, intracellular lipids were stained using Oil Red O. Images were prepared with an Olympus microphotographic system at a magnification of 60×.

**Figure 2:**
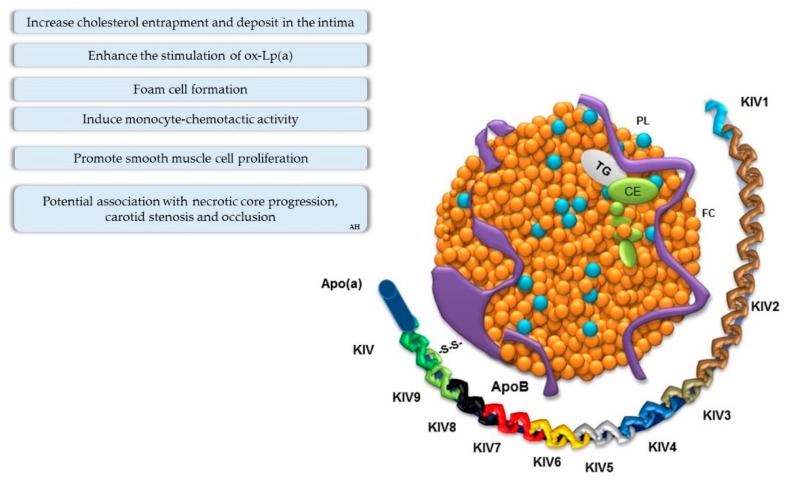
Pathogenic roles and structure of lipoprotein(a) (Lp(a)). Lipoprotein(a) is composed of an LDL-like particle and apolipoprotein(a) (apo(a)), which binds to apolipoprotein B (apoB) through a disulfide bridge. Apo(a) is made up of repetitive amino acid loop-like domains formed by cysteine-rich kringles (so-called kringles (Ks)). PL, phospholipid; FC, free cholesterol; TG, triglyceride; CE, cholesterol ester.

Atherosclerosis is defined as a chronic progressive unresolved inflammation associated with local deposits of cholesterol inside the subendothelial layer of the vascular wall [7,12] (Figure 1 and Figure 2). The starting point of atheroma formation is believed to be an endothelial dysfunction or activation, which is the underlying pathology of cardiovascular disease (CVD). However, earlier studies have proposed that the main factor that dictates the formation of plaques is the response associated to lipoprotein retention within the arterial wall. Potential contributors to early atherogenesis are presented below. 

### 2.1. Lipid Retention

In patients with high blood cholesterol levels (>240 mg/dL), low-density lipoprotein cholesterol (LDL-C) is found to be inversely associated with endothelial-dependent vasodilation [9,13]. In the early steps of plaque formation, it has been suggested that there is a major change in endothelial function where the permeability of atherogenic LDL particles is increased. As detailed in Table 1, this active process leads to foam cell formation that contains cholesterol (Figure 1). This can ultimately form the lipid core of the plaque, which occurs after apoptosis [14]. Analysis of the secretome of vascular smooth muscle cells (VSMCs) in vitro demonstrated that VSMCs are the main source of lipid-retentive extracellular matrix [15]. In more advanced plaques, lipid particles residing in the foam cells are an important contributor to inflammation [16]. This issue is consistent with prior studies that evidenced that cholesterol plays a crucial role in promoting atherosclerosis. However, the endothelial dysfunction and inflammatory aspects of the disease should be kept in mind [3,17]. The molecular and cellular machineries used by lipoproteins that contribute to the genesis of atherosclerotic plaques have not been completely clarified [18].

### 2.2. Plaque Progression

In animal models, the progression of atherosclerosis likely involves effects on VSMC migration, proliferation through the production of factors such as platelet-derived growth factor (PDGF129), and phenotype switching in the lesion core [19]. Advanced lesions show slight VSMC proliferation and VSMC death through apoptosis and necrosis [20] (Table 1). Lineage tracing studies in mouse models with atherosclerosis have revealed extensive proliferation of a low proportion of highly plastic VSMCs [21]. Therefore, at least a subset of foam cells in atherosclerotic plaques is derived from VSMCs [22]. This could contribute both positively and negatively to the progression of the disease. However, it is unknown if VSMCs proliferate and display plasticity or whether individual cells can switch to multiple phenotypes. Furthermore, it is anticipated that a small number of VSMCs contribute to atherosclerotic lesion formation. Indeed, the SMCs within both atherosclerotic lesions originating from a small subset of medial SMC myosin heavy chain Myh11^+^ suggests that SMCs are derived from a single clone [21]. It has been hypothesized that this SMC clone becomes dominant, possibly through epigenetic reprogramming, namely deoxyribonucleic acid (DNA) hydroxymethylation [23,24]. This is supported by the SMC-specific knockout (KO) of the pluripotency factor Oct4 [25]. Data have indicated a marked depletion of SMC within advanced atherosclerotic lesions, which is likely related to the impairment of the migratory capacity of the medial SMC. Thus, various VSMC clones become dominant, but further mechanistic studies are required to clarify the mechanisms by which they are solicited. Therapeutic targeting of these hyperproliferating VSMCs might effectively reduce vascular disease without affecting vascular integrity. In summary, it is becoming clearer that plaque development is a succession of incidents from clinical injury and healing rather than a just gradual process [26]. This idea is obviously supported by the pathological results of different plaque laminations, indicating stepwise plaque evolution [26,27]. However, it remains ambiguous whether there is a single sequence of events during the progression of all lesions [28].

#### Role of Lipoprotein(a)

Lipoprotein(a) (Lp(a)) plays a pathogenic role in the process of atherosclerosis and thrombosis formation [29]. The contribution of Lp(a) to this process is presented in Figure 2. Metanalysis studies and genomic studies have suggested that myocardial infarction (MI), stroke, and calcific aortic valve stenosis are associated with Lp(a) levels [30]. Lp(a) is one of the few risk factors capable of promoting both early and advanced stages of atherogenesis [31]. Indeed, circulating Lp(a) binds to the extracellular matrix in part via its apolipoprotein B component [32], thereby contributing cholesterol to the expansion of atherosclerotic plaque (Table 1). At this stage, the accumulation of native Lp(a) in a vessel’s wall increases cholesterol deposits and may enhance the stimulation of oxidized Lp(a) (ox-Lp(a)), a more potent atherogenic lipoprotein [33]. Further, Lp(a) promotes smooth muscle cell proliferation and induces monocyte–chemotactic activity in subendothelial spaces [34]. This may be related to the promotion of an antifibrinolytic environment and the ability to bind oxidized lipoproteins [35]. In summary, elevated Lp(a) levels may promote atherosclerosis via Lp(a)-derived cholesterol entrapment in the intima, inflammatory cell recruitment, and/or the binding of proinflammatory-oxidized phospholipids. 

## 3. Vulnerable Plaque Controversy

The rupture of atherosclerotic plaque is considered to be the principal mechanism that accounts for (MI) and stroke [1]. Jargon to describe vulnerable plaque has recently become relatively standardized [5,26]. Modern literature often uses this term to denote plaque susceptible to rupture, leading to thrombosis [46]. It is thought that “high-risk” and “thrombosis-prone” are largely used as synonyms to describe “vulnerable plaque” [47]. Retrospective autopsies recommend that there are various histological categories of vulnerable plaque [28]. So-called vulnerable plaques are identified as nonobstructive and silent coronary lesions that suddenly turn obstructive and symptomatic [44]. The combination of a fibrous cap and a large lipid core is known as thin cap fibroatheroma (TCFA), a hallmark of vulnerable plaque [14,28]. Inflamed TCFA is the most shared form of so-called vulnerable plaque and accounts for 60–70% of acute coronary thrombosis events [28]. A coronary thrombosis event is usually qualified as plaque rupture, but further etiologies comprise plaque erosion and calcified nodules [46]. Plaque erosion and plaque rupture are particularly different [48]. Plaque erosion can also arise without the contribution of a lesion’s lipid core [49]. Importantly, about 75% of all acute coronary events result from plaque rupture [50]. Pathology studies have suggested that plaque rupture is associated with 77% stenosis, whereas plaque erosion has an average of 70% area stenosis [51,52]. Mechanically, plaque disruption is expected to happen once the strength of the plaque cap is exceeded by the stresses in the plaque cap [53]. The depth of cholesterol crystal inside coronary plaques has recently been proposed as an index of plaque vulnerability [54]. Various unanswered queries remain, since the pathological description of plaque vulnerability lacks physiological data [42,55]. However, in addition, histological reports are prepared from static and inert tissue, while plaque rupture is a further active process [42,56]. 

## 4. Biochemical and Genetic Markers of Vulnerable Atherosclerotic Plaque

Biological markers of vulnerable lesions are of capital importance in clinical practice. They are proposed to prevent clinical sequelae associated with symptomatic plaque rupture. Various biomarkers related to plaque destabilization are discussed in the following paragraphs.

### 4.1. Biochemical Markers

These are recognized as potential biomarkers for cardiovascular (CV) risk and plaque vulnerability. Complement-reactive protein (CRP) has been consistently reported to induce a prothrombotic state via the induction of tissue factor expression into human monocytes [57]. In addition, CRP predicts the progression of atherosclerosis measured at various sites in the arterial tree [58]. In the Dallas heart study, it was revealed that the CRP level is a poor predictor of atherosclerotic burden [59]. These findings indicated that CRP levels are an important risk factor for CVD progression rather than for plaque rupture. Many cytokines have been implicated in atheroma formation and complications. The exogenous administration of IL-18 in apoE (KO) mice increases atherosclerotic lesions [60]. Conversely, IL-18 inhibition is associated with a marked reduction in atherosclerosis [60], indicating potential benefits for plaque stabilization (Paragraph 7.3). Prospective studies have demonstrated a strong association between serum levels of IL-18 and future CV events in patients with coronary artery disease (CAD) [61]. Other prospective studies have supported findings that elevated fibrinogen levels are associated with an increased risk of fatal CV complications [61]. However, in the Copenhagen City Heart Study, fibrinogen levels could predict advanced atherosclerosis but not rupture-prone carotid plaques [62]. To date, it is still unclear whether high fibrinogen concentrations are a cause or a consequence of atherosclerosis. The soluble suppression of tumorigenicity 2 (sST2) has emerged as a strong prognostic biomarker in patients with heart failure and MI. Indeed, sST2-treated mice developed significantly larger atherosclerotic plaques [63]. In a large cohort of patients with stable CAD, increased sST2 was an independent predictor of long-term all-cause mortality [64]. Pregnancy-associated plasma protein A (PAPP-A) serum concentration was proposed as a marker of unstable atherosclerotic plaques [65]. Interestingly, higher PAPP-A levels were found to be associated with higher TCFA burden in patients with CAD [66]. Therefore, PAPP-A might be a valuable biomarker to predict plaque instability. Additionally, myeloperoxidase (MPO) has been proposed as a major contributor to the formation and rupture of plaques [67]. However, despite various assays for MPO being available for clinical use, its routine measurement has not been suggested in any clinical situations [68]. Importantly, matrix metalloproteinase (MMP) studies have recognized that MMP-2, MMP-8, and MMP-9 are proteases that contribute to plaque rupture and clinical events by degenerating the structural components of the plaque matrix [68,69]. Particularly, MMP-9 plasma concentrations predicted stroke and CV death in patients with ≥50% carotid stenosis [70]. Particularly, the predictive value of MMP-9 was significantly improved when combined with other MMPs (MMP-7 and MMP-8) in carotid artery disease [71]. Other studies have highlighted MMP-9 as a potential therapeutic target for stabilizing rupture-prone plaques in the coronary artery [72]. In a multicenter study, these biomarkers of plaque instability did not seem helpful in the early diagnosis of acute MI, but may have provided some incremental value in the risk stratification for chest pain [73]. 

### 4.2. Genetic Markers

Several genetic association studies have provided new insights into plaque destabilization pathophysiology by identifying new potential biomarkers of CV risk [74,75]. The MMP3 6A6A genotype is suggested to be associated with atherosclerosis, and the 5A allele might play a role in plaque destabilization [76]. There have been several genetic association studies with MMP variants, although the conclusions from many of those were complicated, since they had poor methodology, particularly small sample sizes, and the possibility of publication bias. Various studies have reported the potential proatherogenic role of the *A Disintegrin and Metalloproteinase with ThromboSpondin type 1 repeats* (ADAMTS-7) gene in human carotid atherosclerotic lesions and in human coronary arteries [77]. A study found increased ADAMTS-7 levels in lesions from symptomatic patients [75]. Recent data have identified a tissue inhibitor of metalloproteinase-4 (TIMP-4) as a likely physiological inhibitor of ADAMTS7 [78]. However, it is essential to identify the physiological targets of ADAMTS7 to enable a causal link to be established between ADAMTS7 functions and CAD/atherosclerosis. In the Coronary Artery Risk Development in Young Adults CARDIA study, the *Secreted Phosphoprotein Gene* (SPP1) had a specific association with coronary artery calcification and stroke, [79] with possible implications for plaque instability. However, despite the importance of genome-wide association studies in identifying novel potential biomarkers of CAD, their predictive value is still unclear. 

## 5. Imaging Biomarkers for Vulnerable Plaque 

To date, considerable attention has been focused on developing new approaches to allow a more accurate illustration of the structure and vulnerability of atherosclerotic plaque [26,80]. So far, the methodology of reliable cap rupture risk assessment is still largely debated [54,81,82]. As a reminder, a diagnostic technique’s main objective is to prevent the overtreatment of stable plaques and to guarantee the detection of all rupture-prone plaques [55]. Many diagnostic techniques have provided “promising” findings to detect or predict vulnerable plaques, but with little progress [55]. These include, but are not limited to, computed tomography (CT), spectroscopy, palpography, virtual histology, optical coherence tomography (OCT), high-frequency intravascular ultrasound (IVUS), and magnetic resonance imaging (MRI) [83,84,85,86,87,88]. 

### Limitations and Advantages

Imaging techniques focus on general morphological parameters and are only indirectly connected to the actual cause of plaque severity [26,89]. The IVUS method allows for the direct imaging of vessel wall screening in ruptured plaques and is poor at noncoronary lipid assessment [90]. Although intravascular MRI facilitates the high resolution and morphological characterization of plaques, this method is time-consuming, with potential heat buildup inside the vessel wall [91]. Additionally, OCT is excellent for the penetration of vessel walls and to identify vulnerable plaques with high resolution [92]. However, this approach suffers from various disadvantages [93]. In fact, one of the major limitations is that fatty tissues absorb light and cast a dark shadow behind the tissue, which limits the imaging outside of the necrotic core [93,94]. Indeed, this technique has limited penetration depth [94]. Technically, this may affect the accuracy of imaging and the determination of the geometric features of unstable plaques [95]. Tian et al. [96] showed that fibrous cap depth is the best way to discriminate ruptured plaques from non-ruptured TCFA by combining OCT and IVUS. Moreover, Uemura et al. [97] showed that OCT may potentially provide an even higher predictive value for future events given superior resolution compared to virtual histology IVUS. More prospective studies are warranted to establish the clinical significance of OCT-derived TCFA. Furthermore, the noninvasive CT method is applied in calcified plaque detection, but it does suffer from a lack of clinical experience with plaques and radiation exposure with negative predictive value [98,99]. Additionally, noninvasive MRI methods have been shown to discriminate advanced lesions from early and intermediate atherosclerotic plaques and also identify fibrous cap rupture [100]. According to MRI-based 3D, plaque wall stress values are more closely associated with plaque ruptures than critical flow shear stress [101]. This may indicate that critical wall stress values may become indicators of high-risk sites of ruptures. Some studies have suggested that unstable plaque assessments by CT and MRI methods are potentially useful in improving patient risk stratification and in guiding the use of systemic treatments [102]. It is becoming more recognized now that biomarkers based on MRI models of plaque significance, such as mechanical forces defined as vulnerable plaque wall stress or low shear stress, may be considered during the early prediction of possible acute vascular events [82,103]. Most computer models assume isotropic behavior, while it is well accepted that plaque tissues also perform anisotropic mechanical behavior [104]. The use of imaging modalities has contributed to a better understanding of plaque vulnerability. Nonetheless, the accurate prediction of coronary thrombosis has yet to be accomplished, partly due to the limitations of existing imaging technologies and limited prospective data [46]. Unfortunately, reliable computer modeling of plaques still requires laborious investigations to improve the accuracy of plaque modeling [104,105]. 

## 6. Plaque Rupture Prediction and Challenges

Prior findings have indicated that more than 70% of all CVD events are related to plaque burden [106]. Current outcomes based on clinical scores are not accurate in predicting patients who are at high risk for an acute ischemic event [89]. However, studies on plaque rupture risks have focused on general morphological parameters. These have been limited to the degree of stenosis [88,107], cap thickness [42,108], necrotic core size [95], and the occurrence of hemorrhages in carotid artery plaque [109,110]. Results from these data have revealed that plaques with larger necrotic cores and thin fibrous caps are more susceptible to rupture [14,111]. Particularly, not all destabilized plaques will rupture [5]. The prevalence of silent ruptures in stable CAD is around 58% [112]. Most heart attacks occur in the context of plaque-related thrombus formation [5]. Pathology studies have defined the cutoff thickness of a “thin” fibrous cap as 65 µm [28]. Although a thin fibrous cap less than 65 µm is generally accepted as a vulnerable plaque, some reports have used higher thresholds (>200 µm) to define vulnerable plaques [113,114]. What is unlikely is that vulnerable lesions show mostly a 30% luminal obstruction. It has also been difficult to detect these rupture-prone plaques through conventional imaging modalities [115]. This indicates in part that current rupture risk methods may be unreliable [55]. For better prediction, the sensitivity and specificity of technologies used to detect vulnerable plaques need to be improved. Stress calculation in vulnerable plaques may improve risk prediction [94]. Peak cap stress calculation may help in predicting cap ruptures [94,101]. However, geometric and compositional information obtained via intravascular imaging devices seems to be inadequate to predict CVD events [116]. Therefore, novel rupture risk parameters should be explored [94,116]. To that end, there is a necessity to develop noninvasive methods that can be broadly accessible and accurately stratify a patient’s risk, which would possibly lead to clinical trials. This may refine our capacity to identify high-risk plaques, with important implications for risk prediction and drug development.

## 7. Potential Therapies for Promoting Plaque Regression

Various targets have been proposed to induce plaque regression or slow down the atherosclerosis process. Some potential therapeutic targets are discussed in the following paragraphs within the idea of plaque regression (Table 2).

### 7.1. Evidence of Plaque Regression

The plaque regression concept has been evidenced from earlier animal studies [150] and from patients undergoing medical therapies [151,152]. Clinically, pertinent regression or progression is defined as a change from baseline to follow-up of ≥10% for diameter stenosis [153] and ≥0.2 mm for minimal lumen diameter [154]. Murine apo E^−/−^ or the LDL receptor^−/−^ has suggested that plaque regression can occur [155]. Plaque regression is an important therapeutic target. However, evidence has shown that plaque regression can be accomplished for advanced lesions [150]. Transplantation using a segment of a plaque-containing aorta from hyperlipidemic apo E^−/−^ mouse helped to investigate features of regressing plaques [82]. In vivo data demonstrated a rapid loss of foam cells from premature lesions within 3 days post-transplantation [156,157]. Yet, a great challenge has been the finding of an atherosclerosis model of spontaneous plaque rupture with humanized endpoints such as MI, stroke, and unexpected death. Recently, these features were proposed in apo E^−/−^Fbn1C1039G^+/−^ mice [143]. This model could evaluate potential plaque-stabilizing therapies to better define systemic anti-atherosclerotic factors and their mechanisms. 

### 7.2. Lipid-Lowering Therapy

The administration of hypolipidemic and antioxidant drugs was proposed to prevent the progress of atherosclerosis. Statins are known to decrease CRP, augment the collagen content of atherosclerotic plaque, alter endothelial function, and decrease the inflammatory components of plaque (Table 2). Numerous clinical trials have highlighted the role of statins [117]. Moderate or intensive statin therapy reduces LDL-C [118], promotes atheroma stabilization [45], and induces coronary plaque volume regression [11,117,152]. Statin therapy also reduces fibro-fatty components and increases the dense calcium volume of atheromatic plaque [158]. Statin has also been associated with reduced IP angiogenesis in the carotid artery [119]. The Study of Coronary Atheroma by Intravascular Ultrasound (SATURN) trial showed that more than 60% of rosuvastatin- or atorvastatin-treated patients showed plaque regression [158,159]. Therefore, statins may not affect the instability features of plaque (as reported by meta-analysis studies) [11]. Unfortunately, atherosclerosis continues to progress in up to one-third of patients, despite high statin treatment [160]. This reinforces the need to reduce the “residual risk” of coronary events. 

#### 7.2.1. Anacetrapib

Anacetrapib inhibits the cholesterol ester transfer protein (CETP), thereby increasing high-density lipoprotein cholesterol (HDL-C) and decreasing serum LDL-C levels. This action mimics the genetic heterozygous CETP deficiency state. The use of anacetrapib in patients with atherosclerosis and who are under intensive statin therapy resulted in a lower incidence of major coronary events than the use of a placebo did (ClinicalTrials.gov number, NCT01252953) (Table 2). Anacetrapib trials showed a reduction in plaque progression (mostly by decreasing non-HDL-C) and an improvement in plaque stability [125]. Importantly, imaging studies are expected to evaluate this finding [161].

#### 7.2.2. Probucol

Probucol is a powerful antioxidant with anti-hyperlipidemic activity [127] (Table 2). This drug is thought to stabilize high-risk plaques, perhaps via various pleiotropic functions such as lipid lowering, anti-inflammation, and scavenger receptor suppression [162]. A recent study in patients with CAD (*n* = 300) found that probucol treatment reduces atherosclerotic plaque areas as well as total cholesterol and soluble thrombomodulin levels [128]. Despite these findings, prospective studies are needed to determine whether a combination therapy of probucol with lipid-lowering agents improves vascular outcomes in subjects with CAD. However, probucol reduces HDL-C caused by the activation of CETP [129] and hepatic scavenger receptor class B type I (SR-BI) [163]. Given that, probucol was left by western countries, although it has been used for a long time especially in Japan. 

#### 7.2.3. Therapies against PCSK9

Recent therapeutic advances have been reported through the use of nonstatin therapies (including ezetimibe) and humanized monoclonal antibody technology. Ezetimibe reduces the selective uptake of cholesterol and other sterols by intestinal epithelial cells via a decreasing expression of the Niemann-Pick C1-like 1 (NPC1L1) protein at the apical membrane of enterocytes [164]. Ezetimibe ameliorates endothelial dysfunction and atherosclerosis regression in coronary arteries [126]. Results from an ezetimibe clinical investigation on the regression of intracoronary plaque evaluated by angioscopy and ultrasound (ZIPANGU) in patients with stable CVD have suggested that ezetimibe and atorvastatin are more effective for plaque regression than statin alone [165]. Antibodies against proprotein convertase subtilisin/kexin type 9 (PCSK9) are used to lower LDL in the management of atherosclerotic CVD risk [161,164]. Inhibiting PCSK9 activity currently requires the use of the monoclonal antibodies (mAbs) evolocumab and alirocumab administered subcutaneously every two weeks or monthly. The Global Assessment of Plaque Regression with a PCSK9 Antibody on Top of Statin as Measured by Intravascular Ultrasound (GLAGOV) trial was the first to compare a combination therapy with statin plus evolocumab (also named Repatha) to statin therapy alone [166]. In this study, evolocumab induced atheroma regression (Table 2). Recently the use of evolocumab for secondary prevention seems unlikely to be cost-effective in Canada [167]. Furthermore, alirocumab (also named Praluent) and evolocumab have recently been accepted as treatments for familial and nonfamilial hypercholesterolemia [131,168]. However, more studies are in demand to assess the effects of PCSK9 inhibition on clinical consequences. 

#### 7.2.4. Therapies against Lp(a)

Three therapies with noticeable effects on Lp(a) levels are the object of continuous trials and are discussed below. The PCSK9 inhibitor evolocumab in a phase II trial investigating evolocumab (AMG 145) [132] and alirocumab in a phase II randomized controlled trial [133] lowered Lp(a) significantly by 24.5% to 30%. However, it is not clear why Lp(a) was reduced by PCSK9 inhibitors and not by statins, since both act through the LDL receptor pathway. Antisense RNA (ISIS-APO(a)Rx) directed against apolipoprotein(a) (apo(a)) synthesis in the liver markedly diminished Lp(a) concentrations in humans by 40% [135]. Therapy against Lp(a) is now in phase III trials with TQJ230 (Novartis), with an investigational agent previously known as AKCEA-APO(a)-LRx specifically targeting elevated Lp(a) [134]. Additional therapies have come from Lp(a) apheresis, where Lp(a) decreased on average by 60–70% [169]. In another study, this was associated with 5% coronary atherosclerosis regression in stable CAD patients with high Lp(a) levels [170].

### 7.3. Potential Molecular Targets for Plaque Regression

The activation of macrophage inflammasomes releases interleukin (IL)-1β and IL-18. It also has promoted atherosclerosis and complications in animal models [171]. Inflammasome stimulations may contribute to plaque erosion and thrombosis exclusively in subjects having CVD risks of type 2 diabetes or chronic kidney disease [172]. In addition, the finding of a monoclonal antibody inhibiting interleukin-1β (IL-1β) (called canakinumab) by targeting the inflammatory pathway [136] (Table 2) received solid support. However, in phase II of the trial, the benefits of canakinumab were not detected on plaque inflammation [138]. The outcomes of this phase II trial were proposed to be addressed by the multinational phase III trial Canakinumab Anti-Inflammatory Thrombosis Outcomes Study (CANTOS). Unfortunately, canakinumab was not approved for CV risk reduction from the CANTOS trial [139]. While CANTOS suggested a favorable profile for inflammasome-derived IL-1β in CVD, the magnitude of the advantage was moderate, and there was an excess of infections associated with this therapy [136], perhaps due to decreased neutrophil levels [172]. Recent progress in this field has suggested that molecules upstream of IL-1β secretion, such as NLR Family Pyrin Domain-Containing 3 (NLRP3), mitochondrial cytidine/uridine monophosphate kinase-2 (CMPK2), or caspase-1/11, may provide further therapeutic targets for preventing atherosclerotic vascular disease [172,173]. Another therapy for plaque stability is exploring vascular growth factor angiopoietin-2 (Ang-2) [18]. Ang-2 activity might play a role in the progress of unstable plaque [174]. Ang-2 blockage with antibody MEDI3617 reduced neovascularization and fatty streak progression in hyperlipidemic mice, but did not reduce the size of pre-existing atherosclerotic lesions [2]. Ang-2 therapy has suggested a safety index in various clinical settings [140]. Methotrexate is another drug targeting inflammation that favored the significant regression of aortic plaque and intima areas in cholesterol-fed rabbits [137]. This drug causes the release of adenine nucleotides and adenosine from cells that can engage G protein-coupled adenosine receptors (notably the A2 A receptor) implicated in downstream anti-inflammatory actions [175]. A methotrexate ongoing randomized trial is exploring the effects of immunotherapies on vascular inflammation assessed in individuals with rheumatoid arthritis (clinicaltrials.gov NCT02374021). Furthermore, anti-inflammatory drugs that impair efferocytosis [176] may also present new weapons to slow down the progression and development of CVD, but their detailed mechanisms are still unclear [8].

### 7.4. Angiogenesis Inhibitors

Angiogenesis is closely associated with plaque progression [142,177], where its role in this process may take part in plaque destabilization and thromboembolic acute events [177]. Various compounds have emerged as alternatives to promoting plaque stability by targeting IP angiogenesis. Earlier studies have reported that the antiangiogenic compounds endostatin and TNP-470 reduced atherosclerosis progression in apoE^−/−^ mice [178]. Another agent is ghrelin, a 28-amino acid acylated peptide [179] that was found to inhibit IP angiogenesis in animal models [141]. As reported recently, ghrelin may act by regulating the expression of vascular endothelial growth factor (VEGF) and vascular endothelial growth factor receptor 2 (VEGFR2), reducing monocyte chemoattractant protein-1 (MCP-1) expression at a late stage of atherosclerosis [141,177]. However, the mechanism of action of ghrelin on plaque stability has not yet been largely explored [141]. Anti-angiogenic medications used in clinical trials (mostly anti-VEGF/VEGFR) for anticancer treatments have been associated with a risk for CV adverse effects, and further studies are needed [142,180]. More recently, various compounds have been studied to inhibit IP angiogenesis with mechanisms of action that interfere with angiogenesis [141,142,143] (Table 2). Studies based on a blocking antibody (bevacizumab against VEGF-A, axitinib against VEGF receptor tyrosine kinase, and DC101 against VEGFR-2) have shown potential ability for the treatment of IP angiogenesis and hemorrhage [143]. Few studies targeting neovascularization have been documented [143], possibly because of the absence of relevant animal models. Previous works have shown that the promotion of angiogenesis in myocardial ischemia is a potential strategy [2]. However, angiogenesis promotes atherosclerosis growth in various animal models and probably causes plaque rupture [142,180]. Hence, more consideration should be paid to harmonizing the regulation of angiogenesis in atherosclerotic CVD when using anti-angiogenic medications [180]. 

### 7.5. HDL Biogenesis and Plaque

A meta-analysis of clinical studies reported that atherosclerosis regression as measured by IVUS after decreasing LDL levels was most likely to be achieved when HDL was also significantly increased [118]. Despite this, the sudden rupture of plaque remains the leading reason for acute events [181,182,183]. The role of HDL in plaque regression, although, is still poorly characterized [118]. The infusion of reconstituted HDL in human subjects with acute coronary syndrome [183,184] was found to promote the regression of atherosclerosis lesions over five weeks of therapy [185]. Other studies have linked plaque regression to the use of HDL mimetic peptides [184,186], which target the HDL biogenesis process through increasing apoA-I production or modulating ABCA1 [122,124]. In vivo studies have shown that macrophage cholesterol efflux (CE) mediated by ABCA1/ABCG1 may have an important role in suppressing apoptosis in advanced plaques [187]. Defective HDL capacity in ABCA1-CE was observed in association with progressive atherosclerotic lesions [12]. As we [88,188] and others [189,190,191] have established, an inverse association exists between HDL-CE, carotid plaque instability, and CVD independently of HDL-C levels. Although this has not yet been determined, CE could be an important biomarker in determining who will develop atherosclerosis. However, more recently, serum CE values did not correlate with plaque vulnerable markers in elderly subjects (~80 years, *n* = 59) [192]. One explanation is that CE may play a more protective role in the earlier steps of atherosclerosis [193]. In support of this, CE’s association with CVD events was found to be more evident in younger populations than in others [192]. More studies should be conducted to assess the differences in HDL cholesterol according to atheromatous plaque severity or instability to better evaluate HDL biogenesis as a therapeutic target for atherosclerosis (Table 2).

### 7.6. Current Perepectives for Reducing Atherosclerotic Plaque

New targets in atherosclerosis research have been reported. Some studies have suggested that semaphorin-3A (sema-3A) reduces atherosclerotic plaque progression by enhancing the motility and function of M2 macrophages and that it also regulates foam cell formation in apoE^−/−^ mice [194,195]. Conversely, sema-3E, another semaphorin, is expressed in atherosclerotic plaques and regulates macrophage retention in plaques [196]. Another target for promoting the regression of atherosclerosis is through the activation of the chemokine receptor CCR7-dependent emigration pathway in macrophages [156,197]. Targeting CCR7 activation by statins was found to induce cluster of differentiation 68 (CD68+) released from plaques rather than promote atherosclerotic plaque regression [198]. This indicates a new prospect for reducing atherosclerotic plaques [38]. However, the role of CCR7 in atherosclerosis is more complex. Furthermore, genomic studies have revealed novel targets for anti-atherosclerotic therapy [176]. These include asialoglycoprotein receptor 1, angiopoietin-related protein 4, and inflammatory pathways that damage efferocytosis (CD 47) (Table 2). There is a need to validate the impact of such targets on plaque regression. The adiponectin receptor (AdipoR) pathway in macrophages is of relevance and may represent another target, since decreased AdipoR expression could contribute to plaque instability [199]. Meanwhile, the AdipoR1 or R2 pathway’s distinct effect on plaque instability should be determined. 

#### Locally Applied Therapy for Vulnerable Plaque

Other options exist for local therapy and plaque pacification. Photodynamic therapy (PDT), broadly used in cancer patients, has been explored in plaque regression therapies. This therapy uses a drug called photosensitizer that is taken up by atherosclerotic plaque and is concentrated within macrophages and vascular smooth muscle cells. After photoactivation with a laser or ultraviolet light, the photosensitizer facilitates the production of cytotoxic oxygen radicals that mediate apoptosis [200]. This technology has demonstrated the ability to destroy macrophages and SMCs mechanically without damaging the structural integrity of the vessels [147]. The application of photodynamic therapy is still limited [145], despite safety trials [145,147]. As was stated in a recent review, the pertinence of photodynamic therapy needs further evaluation, and there are major challenges regarding its translation into a clinical reality [146]. Other options include plaque sealing, which is based on the concept that plaques may be intentionally ruptured with angioplasty balloon inflation after intervention [145]. This technology is performed by placing a stent to prevent acute plaque events. However, this concept fell out of favor after the arrival of coronary stenting and a lack of clinical results [145]. Nanoparticle approaches may provide possibilities for safe gene medication, with the goal of attenuating atherosclerosis [201] (Table 2). Silica–gold nanoparticles used for the atheroprotective management of plaques (the NANOM-FIM trial (NCT01270139)) showed a remarkable regression of coronary atherosclerosis [149]. Several important challenges have persisted regarding nanoparticle drug delivery purposes, where each particle arises from a unique set of design criteria and composites [148]. Despite this, nanotechnology is an exciting strategy for effective medication, as evidenced in vivo, by reducing plaque at risk of rupture and changing inflammatory markers. It would be important to know in the near future if these techniques could be associated with improvements in methods of detecting “high-risk plaque” as well as improved stent technology and understanding of so-called vulnerable plaques [55].

## 8. Conclusion

Our knowledge of plaque biology is expanding to a greater extent. The area of vulnerable plaque is receiving more awareness with the arrival of molecular imaging, which allows for better insight into plaque biology. Current advances in both atherosclerosis imaging and lipid-lowering treatment have presented additional questions for future consideration in the setting of reducing plaque with risk of rupture. Despite recent advances in primary prevention and therapeutic technology, treatment of atherosclerosis based on HDL biology remains in preclinical stages. Importantly, more effective efforts should be directed toward developing more reliable models for integrative approaches to plaque assessment. Hence, the use of a combination of imaging and biomarkers (genetic or plasma proteins) may represent an improvement in the early prediction of future vulnerable plaque.

## Figures and Tables

**Table 1:** Summary of atherosclerosis plaque genesis steps and associated consequences.

Steps	Characteristics and Consequences	Ref.
(1) LDL binds to the subendothelial arterial matrix	Retention/accumulation of ApoB-containing lipoprotein in the arterial intima; Lp(a) binding to extracellular matrix; fatty streaks forming in the vessel wall.	[32] [36] [32]
(2) Chemical modification of LDL: oxidation of LDL, acetylated LDL	Activation of endothelial cells lining the vessel wall, damage to endothelial cells and macrophages and maintenance of leucocyte recruitment.	[37]
(3) Recruitment of monocytes–macrophages to the arterial wall	Activated VSMCs secrete proinflammatory chemokines and contribute to the recruitment of monocytes, which differentiate to macrophages. Expression of leukocyte adhesion molecules on the endothelial wall.	[38] [5]
(3.1) Adhesion of inflammatory cells to endothelium surface	VCAM-1 upregulation; migration of monocytes into the intima; monocyte differentiation.	[37]
(4) Uptake of oxidized LDL by family scavenger receptors (TLR, SR-A, CD 63, and others) Accumulation of Lp(a) in the vessel wall	Scavenger receptors bind and then internalize modified LDL into the media. Macrophages phagocytes, consumes LDL, and forms foam cells. Initiate macrophage buildup in the atherosclerotic plaque. Promote cholesterol accumulation in macrophages, forming foam cells and subsequent fatty streaks.	[39] [40] [33]
(4.1) Macrophage accumulation in arterial wall; cholesterol deposition	Crystallization of cholesterol in atherosclerotic plaques; increased ET-1 production and decreased nitric oxide production.	[41] [42] [43]
(5) Fibrous cap formation	Matrix deposition, migration, and proliferation of VSMCs; loss of proteoglycans or collagen; progressive narrowing and hardening of the arteries.	[44]
(5.1) Advanced plaque: Plaque-associated thrombosis	Plaque erosion/rupture.	[45]

LDL, low-density lipoprotein cholesterol; ApoB, apolipoprotein B; VCAM-1, vascular adhesion molecule-1; TLR, toll-like receptor; SR-A, scavenger receptor A; CD63, cluster of differentiation 63; ET-1, endothelin-1; SMCs, smooth muscle cells; lipoprotein(a), Lp(a); VSMCs, vascular smooth muscle cells. Progression of atherosclerotic plaque is highlighted with a red color.

**Table 2:** Therapeutic options in vulnerable plaque. Summary of strategies to stabilize or regress vulnerable plaque.

Pharmacotherapeutic Strategy	Drug	Aim, Effects, and Clinical Phase	Ref.
Inhibit HMG–CoA reductase	Statin	Inhibit cholesterol synthesis; Increase the clearance of LDL-C; Promote plaque regression and reduce IP angiogenesis; Decrease monocyte recruitment to the plaque; Reduce macrophage accumulation in the plaque; Inhibit the production of MMP; FDA approval	[117] [118] [119] [120] [121]
HDL biogenesis	Fibrate RVX-208 HDL mimetic peptides*	Increase apoA-I production and promote the secretion of LDL. Approved; Plaque regression in mice: RVX phase III; Regression of coronary atherosclerosis; No studies	[122] [123] [124]
CETP inhibitors Combination therapy with statins	Anacetrapib	Regress atherosclerotic plaques; Improvement of lesion stability; Phase III	[125]
Cholesterol absorption reduction: inhibition of NPC1L1	Ezetimibe Atorvastatin	Regress atherosclerosis; Reduction of plaque volume; Approved by FDA	[126]
Lipid-lowering by increasing the rate of LDL catabolism	Probucol	May inhibit early stages of cholesterol biosynthesis; Reduce atherosclerotic plaque; Inhibits ABCA1-mediated cellular lipid efflux; Reduce serum HDL-C; In use in Japan, and left by western countries	[127] [128] [129]
(PCSK9) inhibitors with human antibodies	Repatha^®^ Praluent^®^	Regress atherosclerosis; Hypercholesterolemia therapy; Approved by EMA and FDA	[130] [131]
Lp(a)-lowering therapies	Evolocumab Alirocumab AKCEA-APO(a)-LRx Antisense RNA (ISIS-APO(a)Rx)	Phase II trials Phase III trials Phase II trials	[132] [133] [134] [135]
Targeting anti-inflammatory pathway IL-1β inhibition by monoclonal antibodies	Methotrexate Canakinumab	Residual major CV event risk reduced by 15%; May affect vascular disease progression Phase IV ongoing; Data from the CANTOS trial III were not approved by FDA	[136] [137] [138] [139]
Angiopoietin-2 blockage with monoclonal antibodies	Antibodies: MEDI3617	Promote plaque neovascularization in vivo. No toxicity in vivo. Phase I trials.	[2] [140]
Angiogenesis inhibitors	Ghrelin Anti-VEGF/VEGFR: Bevacizumab	Promote plaque stability in vivo. Experimental phase. Controversy in preventing plaque instability. Inhibit IP angiogenesis. Reduce IP hemorrhage: approved.	[141] [142] [143]
Activate RCT	Urolithin B	Increase RCT in foam cells of apoE^−/−^ mice; Experimental phase	[144]
Mechanical regression therapies: Plaque sealing Photodynamic Thermotherapy	Metallic Bioabsorbale Photosensitizer Plasmonic nanoparticles	Mechanically rupture plaque; Lack of clinical data; limited applications despite safety trials; Absence of clinical data; Promote atheroregression below 40%; Phase I safety trial	[145] [146] [147] [148] [149]

HMG-CoA, 3-hydroxy-3-methyl-glutaryl-coenzyme A reductase; LDL-C, low-density lipoprotein cholesterol; HDL, high-density lipoprotein; RVX-208, resverlogix-208; apoA-I, apolipoprotein AI; LXR, liver X receptor; ABCA1, ATP-binding cassette transporter ABCA1; CETP, cholesteryl ester transfer protein; NPC1L1, Niemann-Pick C1-Like 1; PCSK9, proprotein convertase subtilisin/kexin type 9; IL-1β, Interleukin 1 beta; CANTOS, canakinumab anti-inflammatory thrombosis outcomes Study; Ang-2, angiopoietin-2; anti-VEGF/VEGFR, vascular endothelial growth factor (VEGF) and its receptor; IP, intraplaque. * D-4F, L-4F, 6F, 5A, ATI-5261, and ETC-642; FDA, food and drug administration; EMA, European medicines agency.
